# Microbial diversity involved in iron and cryptic sulfur cycling in the ferruginous, low-sulfate waters of Lake Pavin

**DOI:** 10.1371/journal.pone.0212787

**Published:** 2019-02-22

**Authors:** Jasmine S. Berg, Didier Jézéquel, Arnaud Duverger, Dominique Lamy, Christel Laberty-Robert, Jennyfer Miot

**Affiliations:** 1 Institut de Minéralogie, Physique des Mat00E9riaux et Cosmochimie, CNRS UMR 7590, Muséum National d’Histoire Naturelle, Sorbonne Universités, Paris, France; 2 Laboratoire de Géochimie des Eaux, Institut de Physique du Globe de Paris, UMR CNRS 7154, Université Paris Diderot, Paris, France; 3 Unité Biologie des Organismes et Ecosystèmes Aquatiques (BOREA), Muséum National d'Histoire Naturelle, Sorbonne Université, Université de Caen Normandie, Université des Antilles, CNRS, IRD, Paris, France; 4 Laboratoire de Chimie de la Matière Condensée de Paris, Université Pierre et Marie Curie, Paris, France; The University of Akron, UNITED STATES

## Abstract

Both iron- and sulfur- reducing bacteria strongly impact the mineralogy of iron, but their activity has long been thought to be spatially and temporally segregated based on the higher thermodynamic yields of iron over sulfate reduction. However, recent evidence suggests that sulfur cycling can predominate even under ferruginous conditions. In this study, we investigated the potential for bacterial iron and sulfur metabolisms in the iron-rich (1.2 mM dissolved Fe^2+^), sulfate-poor (< 20 μM) Lake Pavin which is expected to host large populations of iron-reducing and iron-oxidizing microorganisms influencing the mineralogy of iron precipitates in its permanently anoxic bottom waters and sediments. 16S rRNA gene amplicon libraries from at and below the oxycline revealed that highly diverse populations of sulfur/sulfate-reducing (SRB) and sulfur/sulfide-oxidizing bacteria represented up to 10% and 5% of the total recovered sequences *in situ*, respectively, which together was roughly equivalent to the fraction of putative iron cycling bacteria. In enrichment cultures amended with key iron phases identified *in situ* (ferric iron phosphate, ferrihydrite) or with soluble iron (Fe^2+^), SRB were the most competitive microorganisms, both in the presence and absence of added sulfate. The large fraction of *Sulfurospirillum*, which are known to reduce thiosulfate and sulfur but not sulfate, present in all cultures was likely supported by Fe(III)-driven sulfide oxidation. These results support the hypothesis that an active cryptic sulfur cycle interacts with iron cycling in the lake. Analyses of mineral phases showed that ferric phosphate in cultures dominated by SRB was transformed to vivianite with concomitant precipitation of iron sulfides. As colloidal FeS and vivianite have been reported in the monimolimnion, we suggest that SRB along with iron-reducing bacteria strongly influence iron mineralogy in the water column and sediments of Lake Pavin.

## Introduction

Ferric iron (Fe^3+^) and sulfate (SO_4_^2-^) reduction together are quantitatively the most important terminal electron accepting processes in both freshwater and marine anoxic environments (e.g., [1–3]). Iron, being the 4^th^ most abundant element in the Earth’s crust, is ubiquitous in freshwater and marine sediments and is therefore greatly exploited by microbial respiration processes there (e.g. [4,5]). Sulfate is the 2^nd^ most abundant dissolved anion in seawater and is often utilized to such an extent that it becomes depleted in the upper centimeters of marine sediments [6]. Competition between microbial iron and sulfate reduction for organic carbon sources and electron donors is governed by thermodynamic yields which are in turn pH- and iron-mineral-dependent (e.g. [7]). Under acidic to neutral conditions predominating in most natural waters, Fe(III) reduction to Fe(II) is expected to be thermodynamically more favorable than sulfate reduction to sulfide (ΣH_2_S). Iron-reducing bacteria have therefore long been thought to outcompete sulfate-reducing bacteria for electron donors, leading to inhibition of sulfate reduction in iron-rich, sulfate-poor zones [8]. However, the dynamics governing the competition between iron- and sulfate-reducing bacteria have recently been re-examined as concomitant iron and sulfate reduction [9,10], as well as the dominance of sulfate reduction over iron reduction [11] have been observed. Even in freshwater systems, where sulfate concentrations are typically 100–1,000 times lower than in seawater, high rates of microbial sulfate reduction can be sustained through rapid re-oxidation of sulfide by sulfide-oxidizing prokaryotes or by abiotic reactions with ferric iron species, and possibly by redox-active organic substances, e.g. certain humic acids [12].

Fe(III)-driven sulfide oxidation produces various reactive intermediate sulfur species, principally elemental sulfur (S^0^), polysulfides (S_n_^2-^), or thiosulfate (S_2_O_3_^2-^) (e.g. [13–15]). These intermediates can then be recycled back to sulfide and sulfate through the activity of sulfur-compound-utilizing microorganisms, including disproportionating bacteria [11,16,17]. This can lead to high turnover of sulfur species (e.g. > 60 times in pure cultures of sulfur-reducing bacteria with ferrihydrite, [18]) mediated by abiotic Fe(III) reduction. Such cryptic sulfur cycling thus deeply impacts carbon, iron, and sulfur biogeochemistry.

In some sulfate-poor freshwaters, sulfur-fueled iron reduction has been shown to be predominant over direct microbial Fe(III) reduction by dissimilatory iron reducers [11]. Sulfate-poor, ferruginous conditions were predominant in the Archean before the Great Oxygenation Event, at the time of Banded Iron Formation (BIF) deposition [19–25]. However, the abiotic and microbial pathways of iron cycling operating under these Archean conditions are still poorly understood [19,26–31]. Modern ferruginous, sulfide-poor meromictic lakes sharing chemical conditions close to those of the Archean ocean provide insights into the dominant microbial and abiotic pathways that may have controlled iron biogeochemistry in the past. Evidence of phototrophic iron oxidation in the ferruginous lakes Matano and La Cruz [32,33] provided a pivotal environmental model for iron oxidation in the ferruginous Archaean ocean. However, the presumed ecological importance of these photoferrotrophs has since been revised. Lake La Cruz photoferrotrophs represent only a minor fraction of the anoxygenic phototrophic population, with the majority apparently supported by sulfur cycling [33,34]. Moreover, recent physiological and genetic evidence suggests that Lake Matano anoxygenic phototrophs are capable of sulfide oxidation under light and sulfide-limited conditions and can thus be sustained by a sulfur cycle, even under ferruginous conditions [35]. It is thus suspected that a cryptic sulfur cycle may be active in these systems and therefore that Fe(III) reduction could be mediated by sulfide in addition to Fe(III)-reducing microorganisms.

In light of these studies, we sought to investigate the microbial potential for iron and sulfur cycling in the high-iron (up to 1.2 mM dissolved Fe^2+^), low-sulfate (< 20 μM) Lake Pavin where iron-oxidizing and -reducing bacteria have been suggested to impact the mineralogy of iron precipitates in the water column and sediments [31,36,37]. Particulate iron is present mostly in the form of amorphous Fe(III)-phosphate at the oxycline, with minor amounts of amorphous Fe(III)-(oxyhydr)oxides [31,36]. With increasing depth, iron is reduced to mixed valence Fe-phosphate, vivianite (Fe^II^_3_(PO_4_)_2_.8H_2_O), and dissolved Fe^2+^ [36]. The occurrence of small amounts of colloidal mackinawite (FeS) has been evidenced by cyclo-voltammetry and formation of greigite (Fe_3_S_4_) is thermodynamically favorable in the monimolimnion [38], both of which result from microbial sulfate reduction. Bacterial sulfate reduction indeed occurs at the redox boundary, but the rather low net sulfate reduction rates (< 2 nmol L^-1^ d^-1^) suggest sulfate limitation [27]. Nonetheless, the full potential for sulfur cycling has likely been underestimated as the recycling of ΣH_2_S by chemical reactions with Fe(III) minerals or biological processes with other electron acceptors (e.g. nitrate) produce sulfur in intermediate oxidation states which can further be microbially reduced, oxidized, or disproportionated (reviewed in [39]).

Previous studies investigating the microbial diversity in the water column of this permanently stratified lake using Sanger sequencing, fluorescence *in situ* hybridization, and cultivation-based methods [40–43], have identified a few Fe- and S-cycling microorganisms. Here, we used high-throughput pyrotag sequencing to obtain a more complete overview of the *in situ* microbial diversity involved in iron and sulfur cycling. By cultivating of some of these Lake Pavin microorganisms on different iron phases identified *in situ*, we investigated how the nature of endogenous Fe species (dissolved Fe^2+^, Fe(III)-phosphate or ferrihydrite), and sulfate availability (S-poor vs. S-rich conditions) influence microbial community composition. Our results suggest that a cryptic sulfur cycle interacting with the biogeochemical iron cycle strongly influences iron mineralogy at the redox transition zone and in the monimolimnion of Lake Pavin.

## Materials & methods

### Geochemistry of the Lake Pavin water column

Samples were retrieved in September 2016, April 2017, and October 2017 onboard a sampling platform anchored over the deepest point of the lake (92 m). Physicochemical parameters (conductivity, dissolved oxygen, pH, and turbidity) were measured *in situ* during each sampling campaign, using a CTD (conductivity temperature-depth) and O_2_-pH-redox probe (YSI 6600), oxygen optodes (nke SDOT) and a turbidimeter (nke STBD 300). Water samples for chemical analyses and culturing were collected with a Niskin bottle at different depths throughout the water column. For the analysis of total dissolved iron (Fe^2+^ + Fe^3+^), water samples were filtered (0.45 μm pore size) and then distributed into vials containing Suprapur HNO_3_ producing a final pH of 1–2. Sulfate and sulfide samples were fixed with zinc acetate to avoid the re-oxidation of any sulfide present. For microbial cultivation, anoxic lake water 10 meters below the oxycline was dispensed into degassed 2L Duran bottles, sealed with butyl rubber stoppers with a N_2_ headspace, and stored at 4°C in the dark until further processing (max. 3 days).

### Enrichment cultures

Sample handling and experiments were performed in a JACOMEX glove box maintained under an Ar atmosphere (Alphagaz 1, Air Liquide, [O_2_] < 10 ppm) to ensure anoxic conditions. All vitamin, trace element, nutrient and buffer solutions were prepared in O₂-free deionized water obtained by Ar bubbling (Alphagaz 1, Air liquide) during 45 min at 80°C. Solutions were sterilized by autoclaving except for the iron and vitamin solutions which were filter-sterilized (0.2 μm).

Enrichments were performed with lake water collected in 2016 to test the impact of different electron acceptors (Fe(III) and sulfate) on iron reduction. Anoxic lake water was buffered with 2-(N-morpholino)ethanesulfonic acid (MES), pH 6.5, and supplemented with 1 mL·L⁻^1^ each of vitamin solution [44], trace element solution [45], and selenite/tungstate solution [44]. A volume of 75 mL of lake water was filled into sterile 100 mL serum bottles with added iron (20 mM Fe provided as ferrihydrite or 10 mM Fe provided as Fe(III)-phosphate or Fe^2+^) and 20 mM Na-lactate and then sealed with butyl rubber stoppers. Cultures to enrich for sulfate-reducing bacteria received an additional 10 mM sulfate provided as Na_2_SO_4_. A summary of all treatment conditions is provided in Table 1. Ferrihydrite was synthesized by adding 1 M KOH dropwise to a 0.1 M Fe(NO_3_)_3_·9H_2_O solution until reaching pH 8 [46]. Nanometer-sized amorphous Fe(III)-phosphate was obtained by the successive addition of 20 mM KH_2_PO_4_ and 20 mM FeSO_4_·7H_2_O to a 0.1 M Na-acetate solution with a pH of 4.6 [47]. Both were prepared under sterile conditions. Fe^2+^ was added from a filter-sterilized 1 M solution of FeCl_2_·4H_2_O.

**Table 1:** Summary of Lake Pavin incubation treatment conditions.

Sample short name	Iron	Organic carbon source	(Additional) e¯ acceptor
FeLS	Fe^2^⁺	lactate	sulfate
FPL	Fe(III)-phosphate	lactate	-
FPLS	Fe(III)-phosphate	lactate	sulfate
FhL	Ferrihydrite	lactate	-
FhLS	Ferrihydrite	lactate	sulfate

Cultures were maintained in the dark at 20°C and periodically sampled with a sterile syringe in a glove box to monitor sulfate and dissolved Fe concentrations. Samples for sulfate and Fe were filtered at 0.2 μm, then fixed as described above. After significant evolution of the culture was observed visually, additional samples were taken for mineralogical and DNA analyses and 5 mL of culture was used to inoculate 40 mL of fresh medium prepared as above except that lake water was filter-sterilized (0.2 μm).

### Chemical analyses

Sulfide species were determined by the methylene blue method of Cline (1969). Sulfate was analyzed on an ion chromatograph (Dionex DX-600 IC System) and concentrations of dissolved Fe were measured by ICP-AES (Perkin Elmer Optima 3000) at the Laboratory of Water Geochemistry, IPGP.

### Mineralogical analyses

The bulk mineralogy of crystalline solid phases formed in enrichment cultures was determined by X-Ray Diffraction (XRD). Samples were collected by centrifugation and rinsed three times in degassed MilliQ water to remove salts before drying on a silicon wafer under Ar atmosphere. Measurements were performed with Co Kα radiation on a PANalytical X’Pert Pro MPD diffractometer in Bragg-Brentano configuration within an anoxic chamber. Diffraction patterns were recorded at 40 kV and 40 mA in continuous-scan mode in the 10 to 100° 2θ range with a 2 θ step of 0.017°. Ten scans of ~1 h each were performed per sample and then integrated and analyzed using the PANalytical X’Pert Highscore software.

Precipitates in enrichment cultures were also analyzed by Scanning Electron Microscopy (SEM). Samples were prepared by filtration through a polycarbonate GTTP 0.2 μm filter (Merck Millipore, Darmstadt, Germany), rinsed with 20 mL of O_2_-free distilled water. After carbon coating, samples were analyzed in a Zeiss Ultra 55 SEM equipped with a field emission gun (FEG) and a Brucker EDX Quantax detector (Brucker Corporation, Houston, TX, USA). Imaging was performed in secondary electron mode (In Lens detector) at 2 kV and a working distance of 2–3 mm. Energy dispersive X-ray spectrometry (EDX) analyses were performed at 15 kV and a working distance of 7.5 mm in backscattered electron mode (SE2 detector).

### DNA extraction and sequencing

For DNA extraction, bacterial cells were concentrated onto 0.2 μm polycarbonate (Millipore) filters mounted in autoclaved Swinnex filter holders and stored frozen at -20°C. Bacterial DNA was extracted using the ZR Fecal DNA Kit (Zymo Research) according to the manufacturer’s protocol, and quantified fluorometrically at 260 nm using the Qubit dsDNA HS Assay KIT (Invitrogen). The extracts were frozen at -20°C until shipping on dry ice to MR DNA (Shallowater, TX, USA) for sequencing. Barcoded amplicon sequencing targeting the hypervariable V3-V4 region (primers 341F and 785R) was performed by MR DNA using bTEFAP technology. In summary, a single-step 30 cycle PCR using HotStarTaq Plus Master Mix Kit (Qiagen, Valencia, CA, USA) was performed under the following conditions: 94°C for 3 minutes, followed by 28 cycles of 94°C for 30 seconds; 53°C for 40 seconds and 72°C for 1 minute; with a final elongation step at 72°C for 5 minutes. Successful amplification was verified by gel electrophoresis. Resulting amplicon products from different samples were mixed in equal concentrations and purified using Agencourt Ampure beads (Agencourt Bioscience Corporation, MA, USA) and sequenced by Illumina MiSeq (Illumina, USA) paired end (2x 300 bp) sequencing.

Sequence data was processed using a proprietary analysis pipeline (www.mrdnalab.com, MR DNA). In short, paired end reads were merged and barcode and primers sequences were trimmed. Short sequences < 150 bp, sequences with ambiguous base calls, and sequences with homopolymer runs exceeding 6 bp were removed. The remaining reads were then denoised, and clustered at 97% sequence similarity. Singleton sequences and chimeras were excluded from analyses. Taxonomic assignment was based on a local nucleotide BLAST [48] search against a curated database derived from GreenGenes, RDPII and NCBI. Between 12·10^3^ and 13·10^4^ sequences were obtained from each sample. The 16S rRNA gene Illumina tag sequences were deposited in the ENA Sequence Read Archive under the study accession number PRJEB26855/ERP108878.

## Results & discussion

### Geochemistry of the lake pavin water column

**Fig 1:**
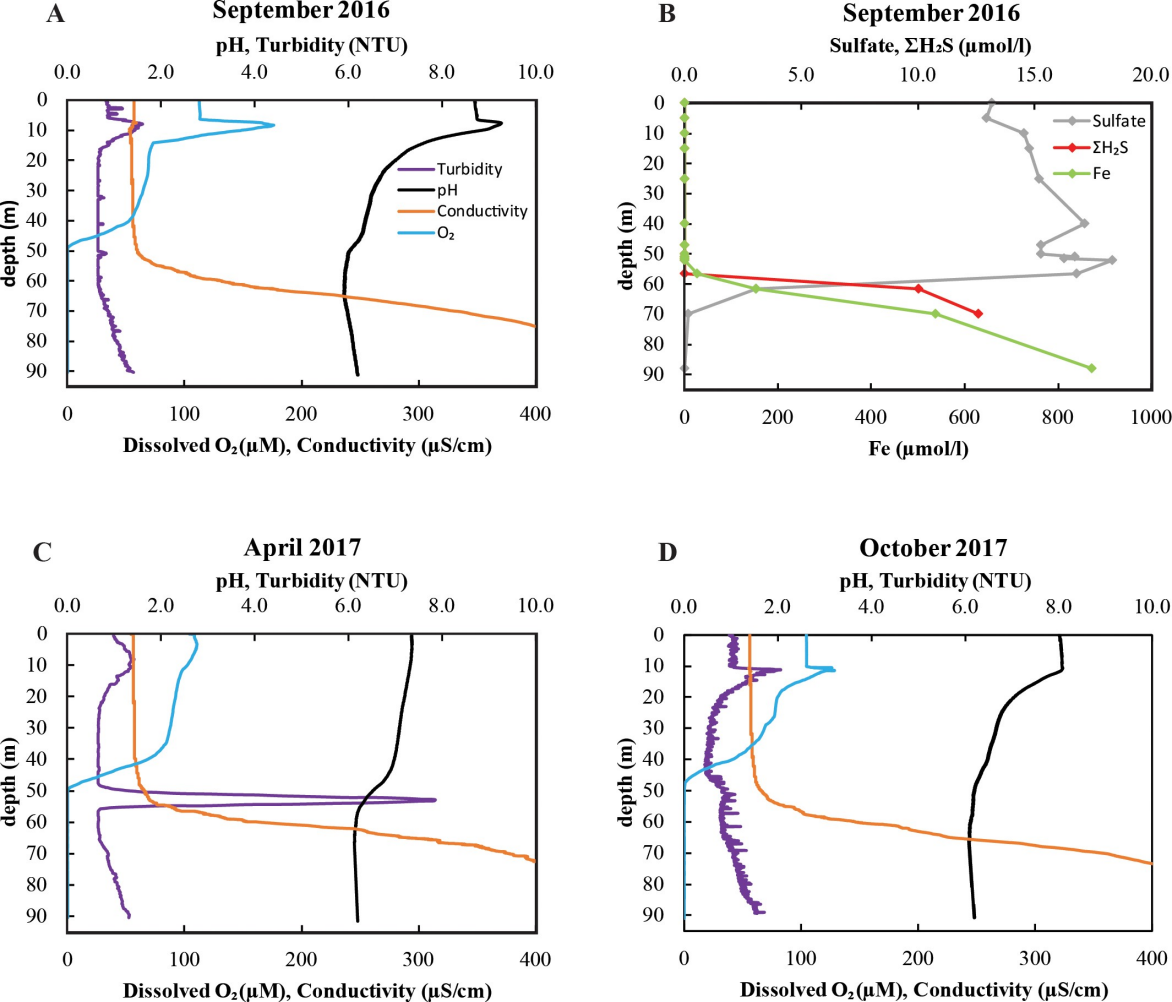
Geochemical profiles of the Lake Pavin water column. Profiles were measured in September 2016 (A&B), April 2017 (C), and October 2017 (D).

As described previously, Lake Pavin is permanently stratified, so that conditions in the deep anoxic water layer (monimolimnion) remain relatively stable from year to year, with slight variations in the position of the oxycline and intensity of the turbidity peak [27,31,38,49,50]. Profiles from the September 2016, April 2017, and October 2017 sampling campaigns were very similar to published profiles (Fig 1). In all years, the maximum gradient for dissolved oxygen was located between 45 and 50 m depth (Fig 1). A maximum of turbidity generally occurs just below the oxycline (Fig 1C), and based on previous studies, this turbidity peak corresponds to massive precipitation of Fe-bearing minerals [36]. The faint turbidity peaks in both September 2016 and October 2017 might be due to a weaker mixing of the mixolimnion and/or a near-complete consumption of oxidants (e.g. O_2_, Mn(IIII/IV), nitrate) earlier in the year. Multiple parameters appear to control the intensity of this turbidity peak (intensity and duration of winter freezing which in turn controls mixing; rates of Fe(III)-mineral sedimentation and reduction; oxidant injection from subsurface springs enhanced by heavy rainfall [51–53]). A nitrate peak of up to 20 μM NO_3_^-^ situated at the oxycline and overlapping with a peak in particulate iron suggests that microbial iron oxidation with nitrate may play a role in iron mineral precipitation [31]. However, only complete biogeochemical modeling would provide a quantification of each contribution to Fe(III) mineral formation. Dissolved iron concentrations increase up to 1.2 mM at the bottom of the lake (Fig 1B & [27,31,38,49,50]). Sulfate concentrations are < 20 μM in the mixolimnion and decrease sharply 10 m below the oxycline coinciding with an increase in ΣH_2_S (Fig 1B & [31]). Cyclovoltammetric analyses have estimated that 80% of this free sulfide is present in the colloidal FeS form [38]. In addition, up to 3.3 μM elemental sulfur has been previously measured in the upper part of the monimolimnion (65 m), then decreasing towards the bottom of the lake [38].

Samples for *in situ* microbial diversity analyses were obtained in October 2017 from 60 m (oxycline), 65 m (oxycline + 5 m) and 70 m depth (oxycline + 10 m) to cover a range of geochemical conditions in the monimolimnion, from microoxic (60 m), to anoxic and ferruginous (65 m), and finally to anoxic, ferruginous and slightly sulfidic (70 m).

### *In situ* microbial diversity

**Fig 2:**
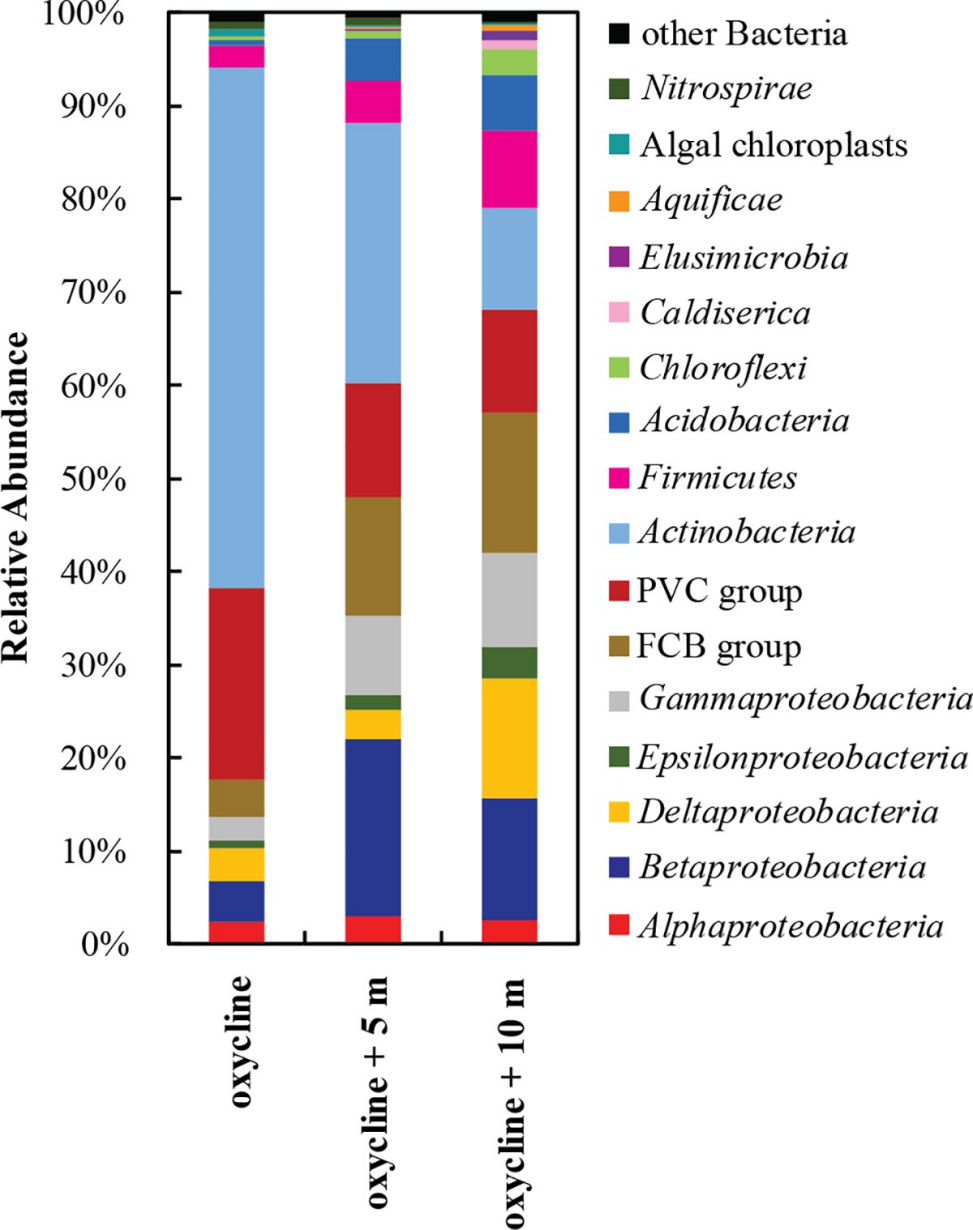
Overview of the total bacterial diversity in the Lake Pavin monimolimnion. Diversity was reconstructed from 16S rRNA gene amplicon libraries from 0 m, 5 m, and 10 m below the oxycline. FCB group = Fibrobacteres-Chlorobi-Bacteroidetes superphylum. PVC group = Planctomycetes-Verrucomicrobia-Chlamydiae superphylum. Clades containing less than 0.5% of sequences were classified as “other bacteria”.

Overall, the diversity represented by our 16S rRNA gene amplicon libraries was consistent with published microbial diversity data from the monimolimnion of Lake Pavin [41,43]. Sequences affiliating with the *Actinobacteria*, *Proteobacteria*, members of the Fibrobacteres-Chlorobi-Bacteroidetes superphylum (FCB group) and members of the Planctomycetes-Verrucomicrobia-Chlamydiae superphylum (PVC group) comprised the majority of sequences at all depths (Fig 2). *Actinobacteria* sequences were most abundant at the oxycline, which is consistent with their predominantly aerobic lifestyle. Conversely, *Proteobacteria* became more abundant with depth, constituting 14, 35, and 42% of all sequences recovered at the oxycline, oxycline + 5 m, and oxycline + 10 m, respectively. Sequences from deeper anoxic waters (oxycline + 10 m) were more evenly distributed into different clades, with a higher representation of deeply diverging taxa such as *Aquificae* (Fig 2). Greater bacterial diversity has previously been observed in anoxic versus oxic waters of Lake Pavin [40,41]. It has been suggested that the absence of competition with O_2_-based metabolisms in anoxic environments allows a higher microbial diversity among microorganisms using anaerobic (i.e. less energetically efficient) metabolic pathways [54].

**Fig 3:**
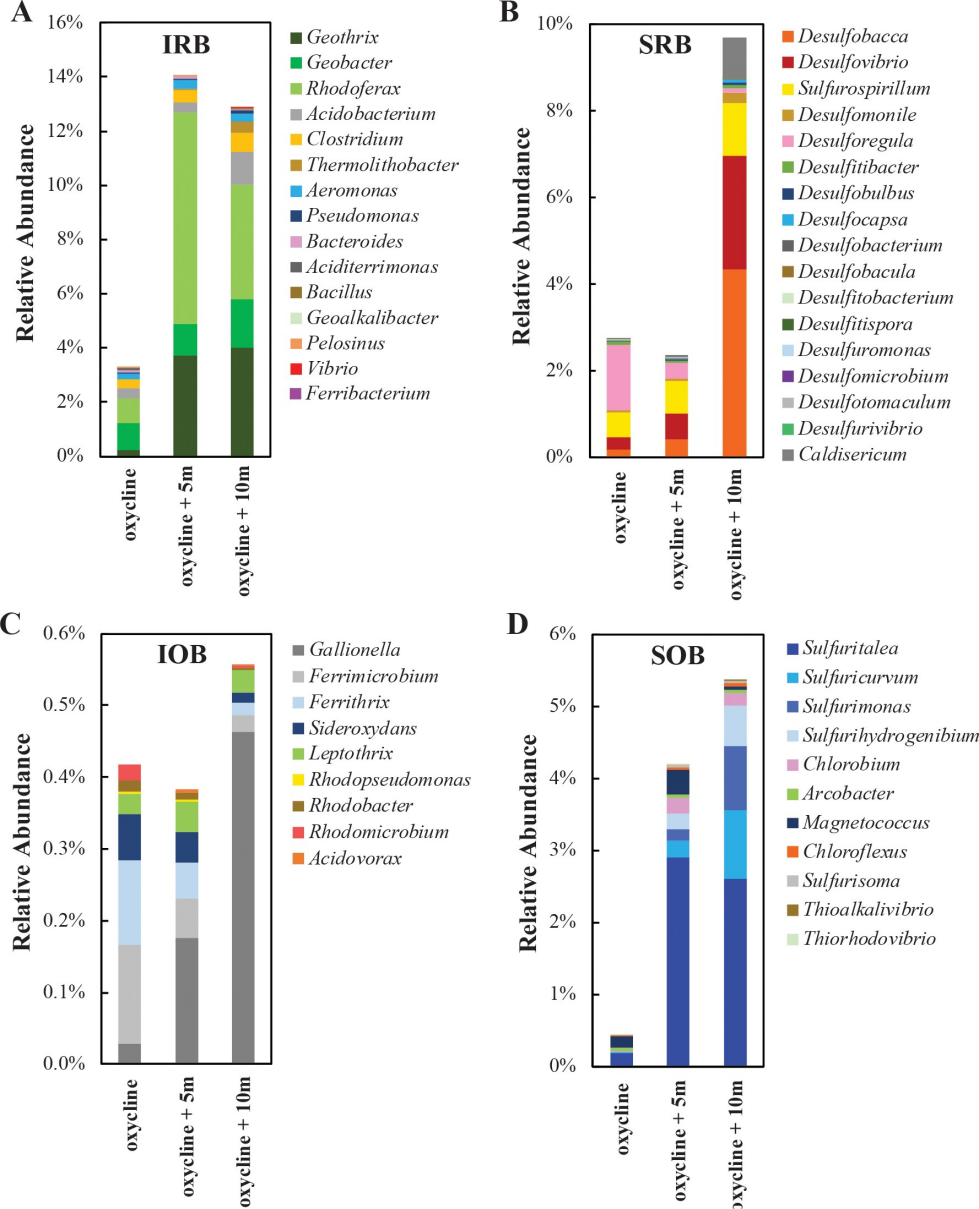
Diversity of iron and sulfur-cycling bacteria in the Lake Pavin monimolimnion. Bacterial 16S rRNA gene amplicon libraries were obtained from 0 m, 5 m, and 10 m below the oxycline. Sequences were classified based on best BLAST hit results, and putative iron and sulfur metabolizing bacteria were identified based on phylogenetic affiliation with (A) iron reducing bacteria = IRB, (B) sulfur/sulfate-reducing bacteria = SRB, (C) iron oxidizing bacteria = IOB, and (D) sulfide oxidizing bacteria = SOB.

We further investigated the metabolic diversity of bacteria in Lake Pavin by identifying putative iron- and sulfur-cycling bacteria based on close sequence affiliation with known iron- and sulfur-cycling microorganisms. Our 16S rRNA gene libraries revealed a large microbial potential for iron and sulfur cycling at and below the oxycline (Fig 3). Approximately 7% of all sequences from the oxycline affiliated with known iron- and sulfur- cycling bacteria and their relative abundance increased to 28% at a depth of 10 m below the oxycline. This trend reflects the geochemical conditions in the lake, where gradients of particulate Fe(III) and sulfate may fuel microbial respiration in the absence of dissolved O_2_. With a few exceptions of rare sequences, most operational taxonomic units (OTUs) were represented at all depths sampled suggesting that the microbial diversity is relatively homogenous across this part of the monimolimnion. However, the full diversity of iron and sulfur cycling bacteria was likely underestimated because some sequences affiliated with groups containing no cultured representatives, and as such their metabolism is not yet known.

Sequences affiliating with known iron-reducing bacteria (IRB) were recovered from all depths sampled but were most abundant 5 m below the oxycline (Fig 3A). The IRB identified comprised both obligate iron-reducers such as *Geothrix* and *Geobacter* as well as facultative iron-reducers, such as *Rhodoferax*, *Aeromonas*, and *Pelosinus*. While other studies have highlighted the importance of facultative (fermentative and sulfate-reducing bacteria) in iron reduction in Lake Pavin [41,42], we found that populations of *Geothrix* and *Geobacter* together constituted a large proportion of IRB at all depths. The most abundant IRB-related sequence retrieved belonged to the purple non-sulfur bacterium *Rhodoferax*, which has previously been detected in Lake Pavin [43]. One of the three cultivated species in this genus, *Rhodoferax ferrireducens*, is capable of dissimilatory Fe(III) reduction with acetate [55]. This microorganism has also been implicated in iron cycling in other stratified lakes [56,57].

Some sulfur/sulfate-reducing bacteria (SRB) are also facultative iron reducers or induce iron reduction indirectly via the production of sulfide. Despite the low concentrations of sulfate in Lake Pavin (< 20 μM) a highly diverse community of SRB was present at all depths sampled and included many genera not yet reported there (Fig 3B). SRB comprised approximately 2.5% of all sequences both at and 5 m below the oxycline and increased to 10% abundance 10 m below the oxycline. *Desulforegula* was the most abundant SRB sequence recovered at the oxycline, but *Desulfobacca*, *Desulfovibrio* and *Sulfurospirillum* became dominant at depth. The variation in SRB community composition may reflect differences in organic carbon or sulfur substrate specialization. For example, *Desulforegula* is known to utilize long-chain fatty acids and long-chain alkenes and is incapable of growth in the absence of sulfate [58], which becomes depleted with depth. In contrast, *Desulfovibrio* are considered important hydrogen utilizers and can also grow on formate, lactate, pyruvate, and many other simple organic compounds [59]. *Desulfobacca* and *Desulfovibrio* can utilize alternative electron acceptors such as elemental sulfur, thiosulfate and sulfite [59,60]. *Sulfurospirillum* was classified as an SRB here because it can reduce elemental sulfur and thiosulfate, but not sulfate [18]. However, *Sulfurospirillum* species have also been observed to oxidize sulfide with nitrate [61,62] and this metabolic versatility may explain its presence across differing redox conditions at all three depths.

Intersecting gradients of oxygen/nitrate and Fe^2+^ at the oxycline provide a potentially excellent habitat for populations of iron-oxidizing bacteria (IOB). About 0.5% of all sequences affiliated with known IOB at all depths sampled (Fig 3C). Interestingly, the obligately aerobic to microaerobic iron oxidizer *Gallionella* [63] comprised 46 and 84% of IOB sequences in anoxic waters 5 and 10 m below the oxycline. This is consistent with previous reports of biomineralized Fe-phosphate stalks reminiscent of *Gallionella* at this depth [31]. As genomic evidence suggests that *Gallionella* are adapted to extremely low oxygen levels, it is possible that they are capable of growth at dissolved O_2_ concentrations below the oxygen detection limits of our sensors (~300 nM) and occupy this narrow niche between O_2_ and Fe^2+^ gradients [64]. Although generally recognized as acidophiles, *Ferrimicrobium* and *Ferrithrix* were also found at the oxycline. Species of these genera are reportedly quite versatile and can oxidize or reduce iron depending on the redox conditions [65]. The only putative denitrifying iron-oxidizers detected belonged to the genus *Acidovorax* [29,66] and were present at very low abundances (< 1% of IOB sequences). Putative phototrophic iron oxidizers *Rhodopseudomonas*, *Rhodobacter*, and *Rhodomicrobium* were most abundant at the oxycline. Previous measurements revealed that only ~0.002% of surface photosynthetically active radiation penetrates to 56 m depth [36], but this is likely sufficient to fuel these low-light adapted anoxygenic phototrophs (e.g. [33,67,68]). Nevertheless, their low abundances (< 10% of IOB) suggest that phototrophic iron oxidation is of relatively low importance in the Lake Pavin water column compared to similar ferruginous lakes such as Lake Matano and Lake La Cruz where abundant populations of anoxygenic phototrophs have been described [32,33]. This is likely due to the high light attenuation by photosynthetic algae in surface waters or precipitation of particulate iron minerals at the oxycline as much less light appears to penetrate below the oxycline of Lake Pavin than the deeper Lake Matano [32,36].

Although at least one green sulfur bacterium (GSB) *Chlorobium ferrooxidans* is capable of oxidizing iron, the role of photoferrotrophic GSB in ferruginous lakes has recently been revised. Because *Chlorobium* in Lake Matano exhibit slow growth and C-fixation rates, and possess a complete set of sulfide oxidation genes, they are thought to subsist mainly by scavenging scarcely detectable sulfide [35]. A recent review of ferruginous environments concluded that even where photoferrotrophs are abundant, sulfur-driven anoxygenic photosynthesis likely overshadows the importance of iron oxidation [34]. Based on these findings, the GSB were classified strictly as sulfide/sulfur-oxidizing bacteria (SOB) here. We recovered sequences affiliating with three genera of phototrophic sulfur bacteria: *Chlorobium*, *Chloroflexus*, and *Thiorhodovibrio* (Fig 3D). These green and purple sulfur bacteria were most abundant at 5 m below the oxycline where they represented only a minor fraction (<6%) of total SOB sequences. This suggests that chemolithotrophic bacteria are the likely key players in the oxidative sulfur cycle of Lake Pavin.

In contrast to a previous study implicating *Epsilonproteobacteria* as the dominant sulfur oxidizers [43], we found that the most abundant SOB in the monimolimnion was *Sulfuritalea*, a member of the *Betaproteobacteria*. The facultatively anaerobic *Sulfuritalea* can grow on thiosulfate and elemental sulfur, but not sulfide [69]. *Sulfurimonas* and *Sulfuricurvum*, which can oxidize sulfide, were more abundant at 10 m below the oxycline, in accordance with the higher concentrations of free sulfide at this depth. Sequences affiliating with *Magnetococcus* were detected in highest abundance at 5 m below the oxycline, where they comprised 8% of SOB sequences. The magnetotactic bacteria of the *Magnetococcaceae* family have been observed to store large amounts of intracellular polyphosphates and are thought to play a role in the phosphorus cycle of Lake Pavin [70]. Overall, SOB sequences increased in relative abundance with depth from 0.4% at the oxycline to 5.5% at 10 m below the oxycline, likely paralleling the increasing concentration of reduced sulfur species (Fig 1B, [38]).

The high relative abundance of SOB and SRB, particularly those capable of reducing sulfur intermediates, at depths where sulfate has decreased to detection limits suggests that microorganisms could drive high levels of sulfur recycling in this low-sulfate lake. While it has been postulated that ferric iron catalyzes sulfide oxidation in ferruginous waters [38,71], a plethora of SOB in Lake Pavin may compete with abiotic reactions consuming reduced sulfur compounds. Some SOB are even capable of oxidizing FeS (e.g. [72]), which could explain the labile nature of colloidal FeS detected by voltammetric measurements [38] but has never been directly observed, e.g. by electron microscopy [31].

### Fe & S-substrate-specific diversity in enrichment cultures

To further investigate the iron- and sulfur-reducing capacities of bacteria found in the monimolimnion (10 m below the oxycline) of Lake Pavin, we enriched for these microorganisms from water collected in Sept 2016 on different Fe minerals that are predominant in the monimolimnion (ferrihydrite = Fh, nanometer-sized amorphous Fe(III)-phosphate = FP) or on dissolved Fe (Fe^2^⁺), with (10 mM) or without (<20 μM) addition of sulfate, and with lactate as the electron donor (Table 1). Lactate was provided at 20 mM, which is much higher than the *in situ* total dissolved organic carbon concentration (0.1–0.5 mM in the monimolimnion [73]) but allowed us to enrich Fe and S cycling bacteria without carbon limitation within a reasonable timeframe. These cultures were monitored for visual changes indicative of growth (S1 Fig) before sampling for mineralogical and diversity analyses. The formation of black precipitates in cultures with added sulfate and FP or Fe^2+^ (FPLS & FeLS) was observed after only 18 days. The culture with FP and no added sulfate (FPL) appeared to evolve somewhat more slowly, turning first dark gray after 49 days, and then blueish after 68 days of incubation. Cultures with Fh both with and without sulfate (FhLS & FhL) turned dark brown and magnetic after about 68 days.

**Fig 4:**
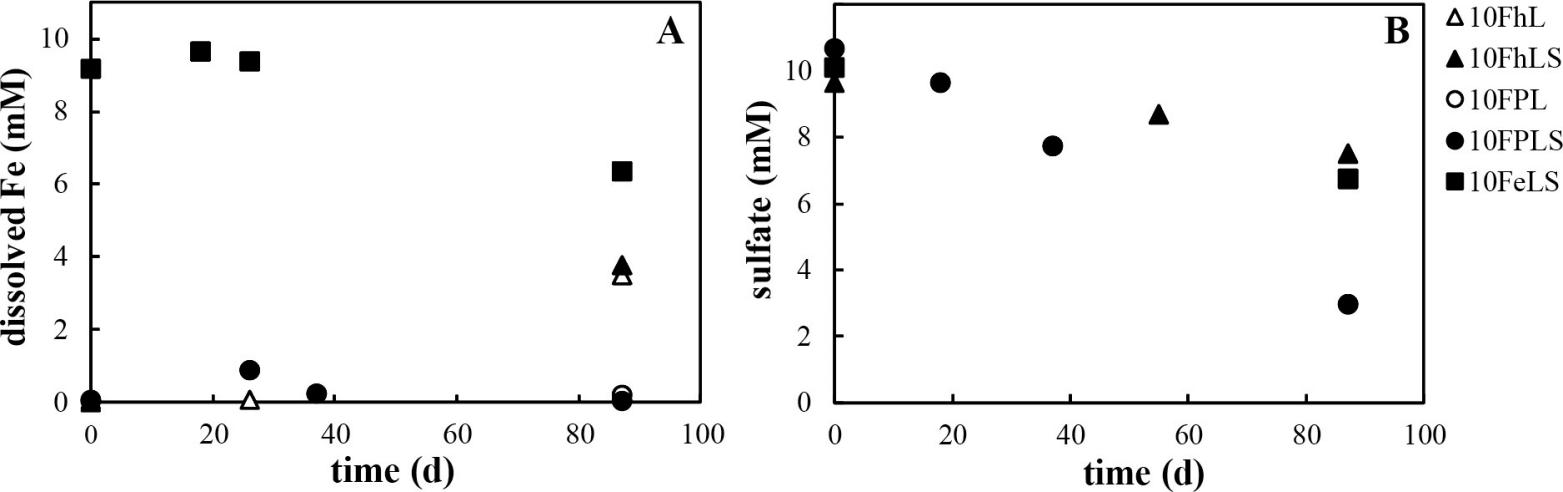
Dissolved iron and sulfate concentrations in Lake Pavin enrichment cultures. (A) Dissolved Fe was measured in cultures amended with different iron phases (ferrihydrite, Fe(III)-phosphate, or Fe^2^⁺), lactate, and with or without added sulfate. (B) Sulfate consumption was monitored in cultures with added sulfate only.

Microbial iron and sulfate reduction in the presence of ferric iron minerals is expected to release soluble Fe and, when excess sulfide is present, concomitantly precipitate Fe-S minerals. Dissolved Fe concentrations increased in Fh-amended cultures FhL and FhLS, from ~ 0 mM to 3.5 and 3.8 mM, respectively (Fig 4A). In contrast, very little dissolved Fe was detected in FP-amended cultures, and dissolved Fe concentrations decreased by approximately 2.8 mM in culture FeLS (Fig 4A). In both cases, the trapping of dissolved Fe can be attributed to the precipitation of secondary Fe-phosphate and/or Fe-S minerals. Precipitation of Fe-S minerals is consistent with sulfate reduction measured in incubations with added sulfate. The consumption of sulfate varied somewhat with the type of iron added, with approximately 70, 34, and 22% of the initial sulfate consumed in cultures FPLS, FeLS, and FhLS, respectively, after 87 days of incubation (Fig 4B). This is evidence that an opportunistic community of SRB thrives in the Lake Pavin water column, despite extremely low sulfate concentrations. It is nonetheless curious that sulfate reduction was much more rapid in culture FPLS than in FhLS and especially FeLS where sulfate was the only electron acceptor. One possible explanation for this is that SRB in incubations FhLS and FeLS were limited in phosphate, a key element in the sulfate activation step to APS. Although phosphate concentrations are relatively high (100–150 μM) in the Lake Pavin water column [27], dissolved phosphate in culture FhLS may have been scavenged by adsorption to the added ferrihydrite, thus becoming unavailable to SRB. The nanometric FP, on other hand, could have served as a net source of phosphate during reductive dissolution of the mineral, thus promoting growth of SRB.

**Fig 5:**
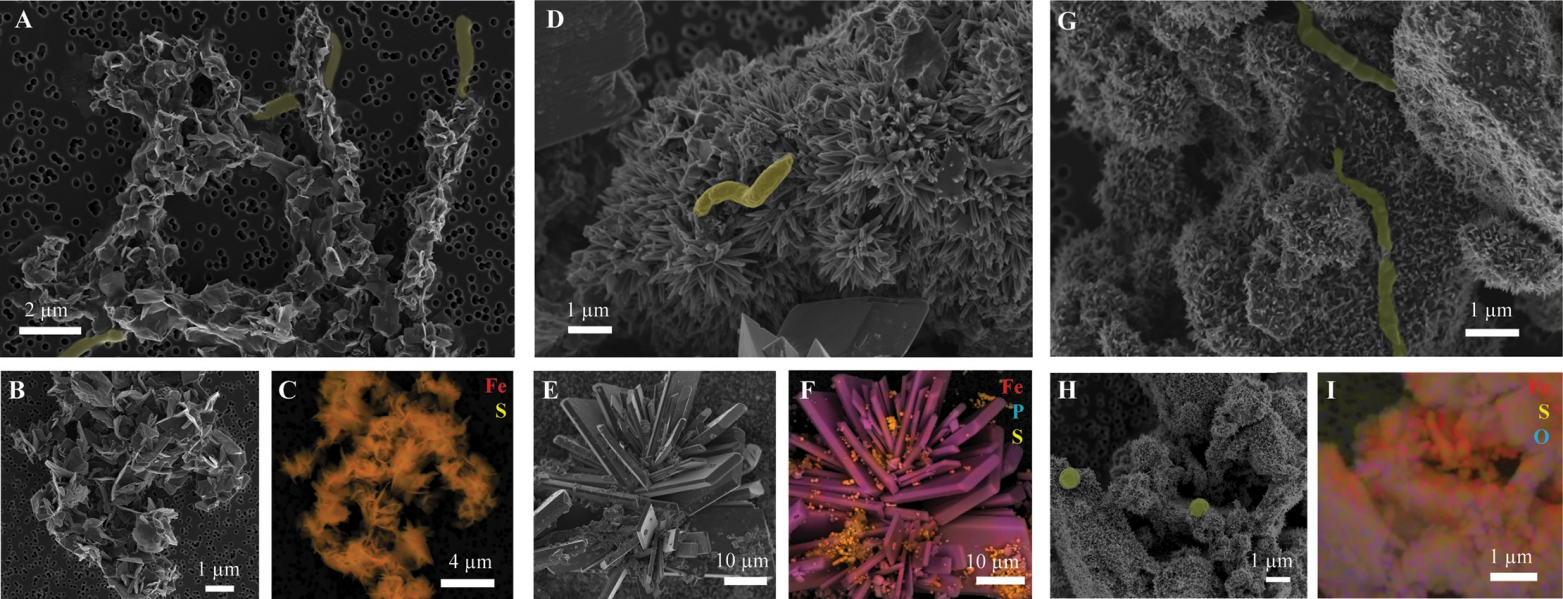
SEM images of morphologically diverse mineral precipitates from Lake Pavin enrichment cultures with different added iron phases. Anoxic lake water from Sept 2016 was incubated with Fe^2^⁺ (A,B), ferrihydrite (D,E), and Fe(III)-phosphate (G,H), with added sulfate and lactate. Corresponding EDX maps (C,F,I) reveal differences in composition of precipitates from each enrichment. Microbial cells are highlighted in yellow. Analyses were performed after significant evolution of the original added iron phase was visually observed: FeLS = 18 d, FPLS = 50 d, FhLS = 75 d.

Mineral precipitates in our cultures exhibited a wide variety of morphologies, from small angular crystals reminiscent of mackinawite (Fig 5A–5C), to larger flattened crystals reminiscent of vivianite (Fig 5E and 5F). The presence of microbial cells encrusted in (Fig 5A) or visibly associated with (Fig 5D, 5G and 5H) these iron minerals suggests that microorganisms play a role in their nucleation and/or transformation.

**Fig 6:**
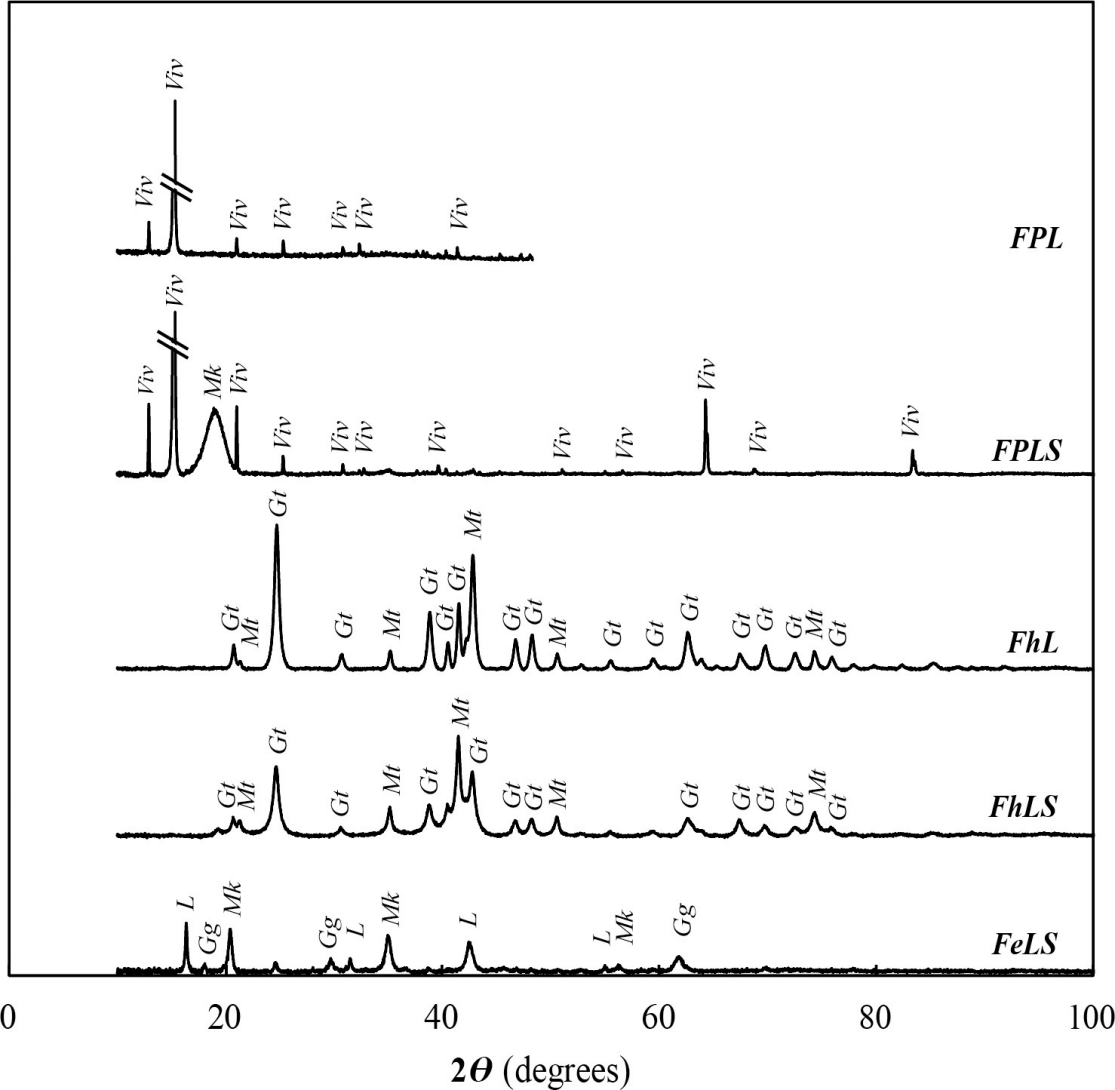
XRD analysis of mineral products obtained in Lake Pavin enrichment cultures with different added iron phases. Anoxic lake water was incubated with Fe^2^⁺, ferrihydrite or amorphous Fe(III)-phosphate, with or without sulfate, and lactate. Viv: vivianite, Mk: mackinawite, Gg: greigite, Gt: goethite, Mt: magnetite, L: lepidocrocite. Hatches indicate where high-intensity peaks have been truncated. Mineralogical analyses were performed after significant evolution of the original added iron phase was visually observed: FeLS = 18 d, FPL & FPLS = 50 d, FhL & FhLS = 75 d.

Further analysis by XRD revealed that the type of mineral formed in each incubation depended on the iron precursor mineral added. In FP-amended incubations, vivianite was the main mineral product formed (Fig 6). Some iron sulfides like mackinawite (Fig 6) formed as well in the incubation with sulfate (FPLS) and were associated mainly with Fe-phosphate mineral surfaces (Fig 5F). These mineral products are in accordance with the dominant Fe(II)-bearing phases observed in the monimolimnion of Lake Pavin [31,36,38], suggesting that iron reduction pathways in our FP incubation are most similar to those occurring *in situ*.

In incubations with Fh, mineral products consisted of Fe (oxyhydr)oxides, namely goethite and magnetite (Fig 5G–5I, Fig 6). Here, goethite and magnetite likely formed via the adsorption of bacterially generated ferrous iron with the ferrihydrite surface and subsequent dissolution/reprecipitation of goethite or solid-state conversion to magnetite [74]. While at least 20% of the initial ferrihydrite was reduced and released as dissolved Fe, the predominance of ferric iron minerals suggests that early formation of these less soluble, less reactive iron minerals may have impeded further iron reduction. The adsorption of phosphate and organic carbon (e.g. humic substances) to iron (oxyhydr)oxides may also have altered their surface reactivity or blocked access to microorganisms [75,76]. Together this could explain the slower evolution of Fh-amended cultures compared to other treatments. Since goethite has never been identified *in situ* and magnetite has only been found in the form of intracellular biominerals in magnetotactic bacteria in Lake Pavin [70], the reduction pathways occurring in the Fh-amended cultures are not likely to be significant in the lake.

In culture FeLS containing added Fe^2+^ and sulfate, well-crystallized mackinawite and greigite were recovered (Fig 6). These Fe-S minerals precipitated from the reaction of microbially-generated sulfide with Fe^2+^ which is likely a relevant process in the Fe^2+^-rich sulfate reduction zone of Lake Pavin where water is supersaturated with respect to mackinawite and greigite [38]. The presence of mineral-encrusted cells (Fig 5A) in our incubation suggests that SRB enhanced mineral precipitation via localized sulfide production or that cell surfaces served as mineral nucleation templates. In addition, the presence of the iron oxyhydroxide lepidocrocite (Fig 6) suggests that some oxidation of iron occurred under sulfur-reducing conditions though this ferric iron phase appeared to be relatively minor (3%; S1 File). Since our incubations were performed under strictly anoxic conditions, it is possible that denitrifying microorganisms utilized endogenous nitrate, present at concentrations of ~ 5 μM at the water depth used for incubations [31,77], to oxidize Fe^2+^ [63].

**Fig 7:**
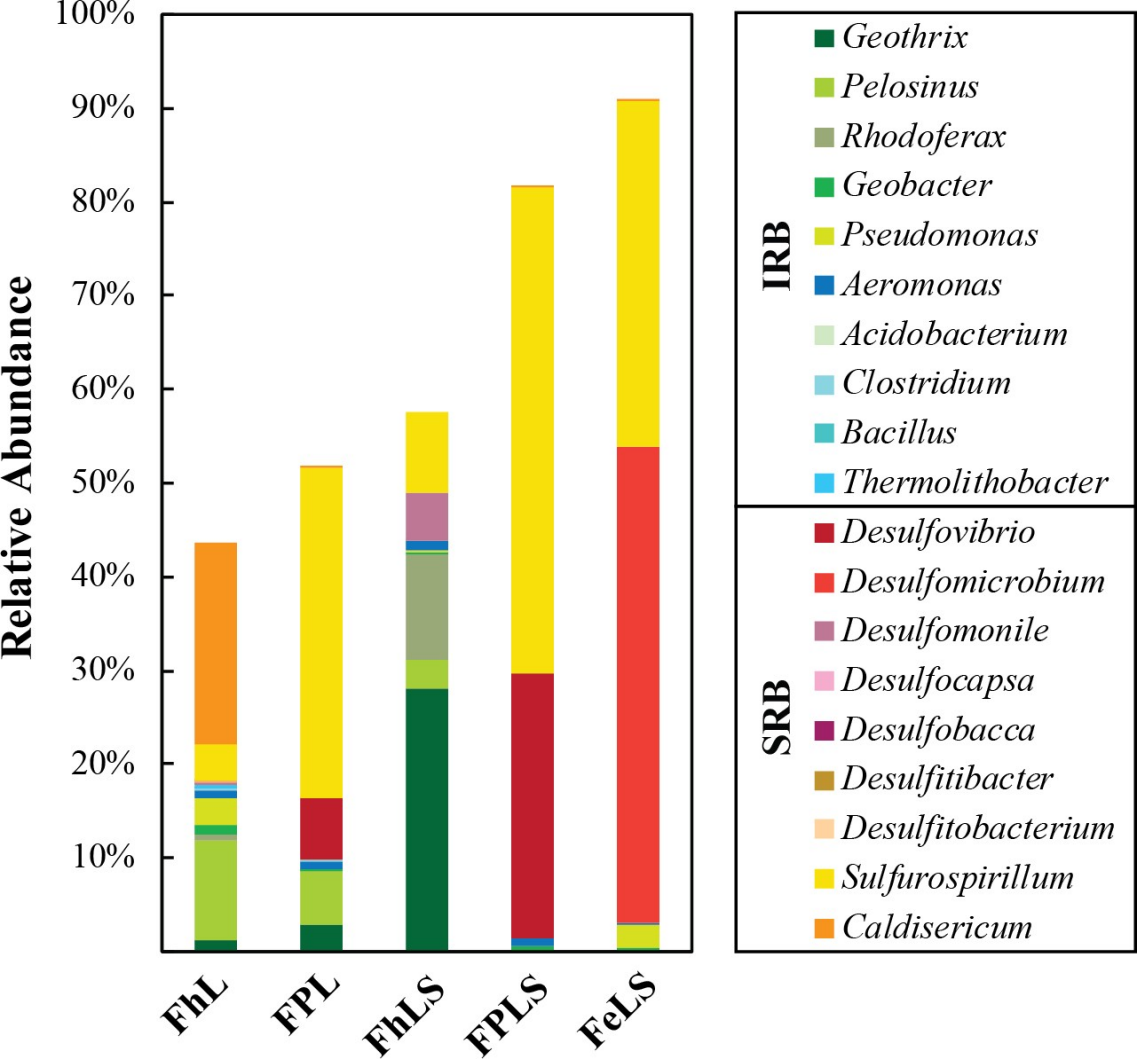
Diversity of iron- and sulfur/sulfate-reducing in Lake Pavin enrichment cultures with different Fe phases. Bacterial 16S rRNA gene sequences obtained from 2016 Lake Pavin enrichment cultures were classified based on best BLAST hit results, and putative iron- and sulfur/sulfate-reducing bacteria were identified based on phylogenetic affiliation with known iron and sulfur/sulfate reducers. Cultures were sequenced after significant evolution of the original added iron phase was observed: FhL = 360 d, FPL = 82 d, FhLS = 55 d, FPLS = 180 d, FeLS = 27 d.

After allowing for significant growth (25 to 365 days, depending on the culture condition), we sequenced the bacterial 16S rRNA gene from each of these enrichment cultures. Our amplicon libraries revealed substantial differences in bacterial community composition across enrichment conditions, suggesting a high degree of metabolic specialization for different iron substrates and sulfur compounds. In general, putative iron- and sulfur- reducing bacteria were highly enriched, comprising 44–91% of all sequences recovered from the enrichments (Fig 7).

Surprisingly, a high abundance of SRB sequences were recovered from cultures without added sulfate, constituting 26 and 42% of sequences from cultures FhL and FPL, respectively. These sequences belonged mainly to *Sulfurospirillum*, *Caldisericum*, and *Desulfovibrio*. Of these genera, only members of the *Desulfovibrio* have thus far been shown to enzymatically reduce Fe(III) [78]. However, *Desulfovibrio* constituted only a minor fraction of the SRB population in FPL and were not detected in cultures FhL. *Sulfurospirillum* and *Caldisericum* are not known to reduce sulfate and presumably thrived on oxidized sulfur intermediates produced by SOB or by iron-catalyzed sulfur cycling, whereby the reaction of ferric iron with sulfide regenerates oxidized sulfur intermediates (e.g. [18]). As only <0.7% of sequences affiliated with known SOB in culture FPL, iron-catalyzed sulfur cycling was most probably predominant in this condition. In contrast, up to 10% of sequences in culture FhL affiliated with the genus *Arcobacter* which may have performed sulfide oxidation with nitrate or nitrite [79], although these compounds were not quantified in our cultures.

In cultures with added sulfate, SRB appeared to dominate the microbial community except for in the condition with added ferrihydrite (FhLS; Fig 7). The low abundance of sequences affiliated with SRB, namely *Desulfomonile* (5%), in culture FhLS is consistent with the relatively low consumption of sulfate observed (Fig 4B). Another 9% of sequences in this culture were closely affiliated with the sulfur-reducer *Sulfurospirillum*. Although the ability to reduce sulfate is not recognized for *Sulfurospirillum*, more than a third of sequences in the culture with sulfate as the only electron acceptor (FeLS) affiliated with this genus. Since no ferric iron was added (and lepidocrocite was present in very low proportion < 5%; S1 File) which could catalyze the oxidation of sulfide to S^0^, it is possible that *Sulfurospirillum* survived on sulfur intermediates produced by SOB in this culture. In fact, a number of facultatively anaerobic SOBs including *Arcobacter*, *Sulfuricurvum*, *Sulfuritalea*, and *Sulfurimonas* were detected, although together they accounted for less than 0.5% of the total recovered sequences. A large proportion of the remaining sequences (51%) in the Fe^2+^ culture belonged to the strictly anaerobic sulfate-reducing *Desulfomicrobium*, which was absent from other cultures. Overall, it is interesting that such distinct communities of SRB developed in the presence of different iron phases at both low and high sulfate concentrations. This demonstrates that these SRB are extremely niche-specialized, not only in their preferences for different sulfur compounds, but also likely for electron donor/carbon sources or chemical conditions (e.g. phosphate availability) enabling the coexistence of a wide diversity of SRB in the sulfate-limited monimolimnion of Lake Pavin.

Putative IRB comprised 18 and 10% of all sequences recovered from the sulfate-free culture FhL and FPL, respectively, and were most abundant (44%) in the sulfate-amended culture FhLS. It is not clear why the addition of sulfate favored the growth of IRB in this case, but it is possible that sulfur compounds served as electron shuttles for these bacteria. The release of comparable amounts of dissolved iron (Fig 4A) together with the dominance of IRB in FhL and FhLS (Fig 7) indicated that dissimilatory iron reduction in this condition was at least as important as abiotic reduction with sulfide. The obligate iron-reducing *Geothrix* was the most abundant IRB detected in FhLS (28%) and was also present in lower abundances (> 3%) in FhL and FPL. *Geobacter* were also detected in these cultures, but in much lower abundances (< 1%). The fermentative bacteria *Pelosinus*, which has commonly been found to dominate electron acceptor-limited enrichments with lactate as the main carbon source and electron donor [80,81], and *Rhodoferax* constituted most of the remaining IRB sequences in these cultures. These results suggest that the nature of the iron substrate, e.g. insoluble minerals, determined the diversity of the IRB community. Whereas transfer of electrons to ferric iron by *Geobacter* spp. requires direct contact with the cell [82,83] or with nanowires [84], iron reduction by *Geothrix* spp. or fermentative bacteria such as *Pelosinus* and *Rhodoferax* may be mediated by soluble electron carriers or enzymes [85,86]. Moreover, facultative iron reducers obtain most of their energy from fermentation of organic substrates, utilizing iron only as a sink for extra electrons [87]. These metabolic differences may also explain why *Geobacter* constitute only a fraction of total IRB in the Lake Pavin water column (Fig 3A) where their habitat is likely limited to the ferric iron particulate surfaces.

Overall, some of the putative IRB and SRB identified in the Lake Pavin water column were successfully enriched on representative Fe compounds both with and without sulfate suggesting that these bacteria are indeed active *in situ*. It also appears that different Fe(III) mineral phases favored the growth of distinct bacterial communities either directly or indirectly (possibly by limiting phosphate availability), and that mineral products depended largely on the initial Fe(III) phase added. Since the mineralogy of enrichments amended with FP evolved in the same way as observed in the lake water column (FP reduction to vivianite and mackinawite) they may serve as model systems for understanding the mechanisms of Fe and S cycling operating in the lake. In our experiments, vivianite precipitated from PO_4_^3-^ and Fe^2+^ released either from microbial reduction of Fe(III)-phosphate or from FP reaction with biogenic sulfide. These data support the hypothesis that microbial iron- and sulfur-reducing processes contribute to vivianite formation *in situ* [36].

## Conclusions

In this study, we identified the potential for microbial iron and sulfur cycling likely influencing the iron mineralogy at the redox transition zone of Lake Pavin. While it is difficult to distinguish between the relative contributions of abiotic (sulfide-driven) iron reduction versus dissimilatory iron reduction, our data show that there is a great potential for microbial iron-reducing activity in the lake water column. Compared to other stratified lakes where phototrophic iron and sulfide oxidation has been described (Lake Matano, Lake La Cruz, Lake Cadagno), Lake Pavin appears to be relatively poor in phototrophic IOB and SOB which constituted only a tiny fraction (< 0.3%) of our 16S rRNA gene libraries. Instead, the identification of microaerobic to anaerobic, chemotrophic IOB *in situ*, together with the formation of the iron oxide lepidocrocite in one enrichment culture, suggests that microbial iron oxidation with oxygen or nitrate may influence iron mineral transformations in the water column. Since nitrate reduction appears to occur just below the oxycline [77], Lake Pavin may be a promising environment for the targeted cultivation and isolation of nitrate-dependent Fe(II)-oxidizers which have not yet been identified in water column environments.

Recent studies have suggested that the importance of microbial iron cycling in iron-rich, low-sulfate lakes may be overshadowed by intensive but cryptic microbial sulfur cycling [34]. The detection of abundant SRB and SOB *in situ* and in our enrichment cultures suggests that sulfur cycling is also highly active in the sulfate-poor waters of Lake Pavin. Moreover, the dominance of obligate sulfur-reducing bacteria in enrichment cultures without added sulfate suggests that sulfur recycling is at least in part driven by re-oxidation of sulfide by Fe(III) minerals forming sulfur intermediates (e.g. S^0^, S_2_O_3_^2-^, and SO_3_^-^), and consequently, that sulfur cycling activity is much greater than previous sulfate reduction rate measurements [27] would suggest.

Based on the transformation of iron mineral phases in our enrichment cultures, we suggest that sulfate and sulfur-reducing bacteria along with iron-reducing bacteria strongly influence iron mineralogy in the water column and sediments of Lake Pavin. Future investigations will elucidate the biomineralization pathways involved in the formation of major iron mineral phases there.

## Supporting information

S1 Fig Evolution of bacterial cultures enriched from the monimolimnion of Lake Pavin. Enrichments were prepared from Lake Pavin water collected in Sept 2016 from 10 m below the oxycline with different iron phases (ferrihydrite = Fh, Fe(III)-phosphate = FP, or Fe^2^⁺ = Fe), without or with 10 mM added sulfate (S), and with 20 mM lactate (L) as an electron donor.
(TIF)

S1 File Fe K-edge EXAFS analysis of enrichment culture with added lactate, dissolved Fe(II) and sulfate (FeLS). (PDF)
