# Supplementary figures and images for: Microbial diversity involved in iron and cryptic sulfur cycling in the ferruginous, low-sulfate waters of Lake Pavin
Source: PLoS One. 2019 Feb 22;14(2):e0212787. doi: 10.1371/journal.pone.0212787 (PMC6386445; doi:10.1371/journal.pone.0212787)

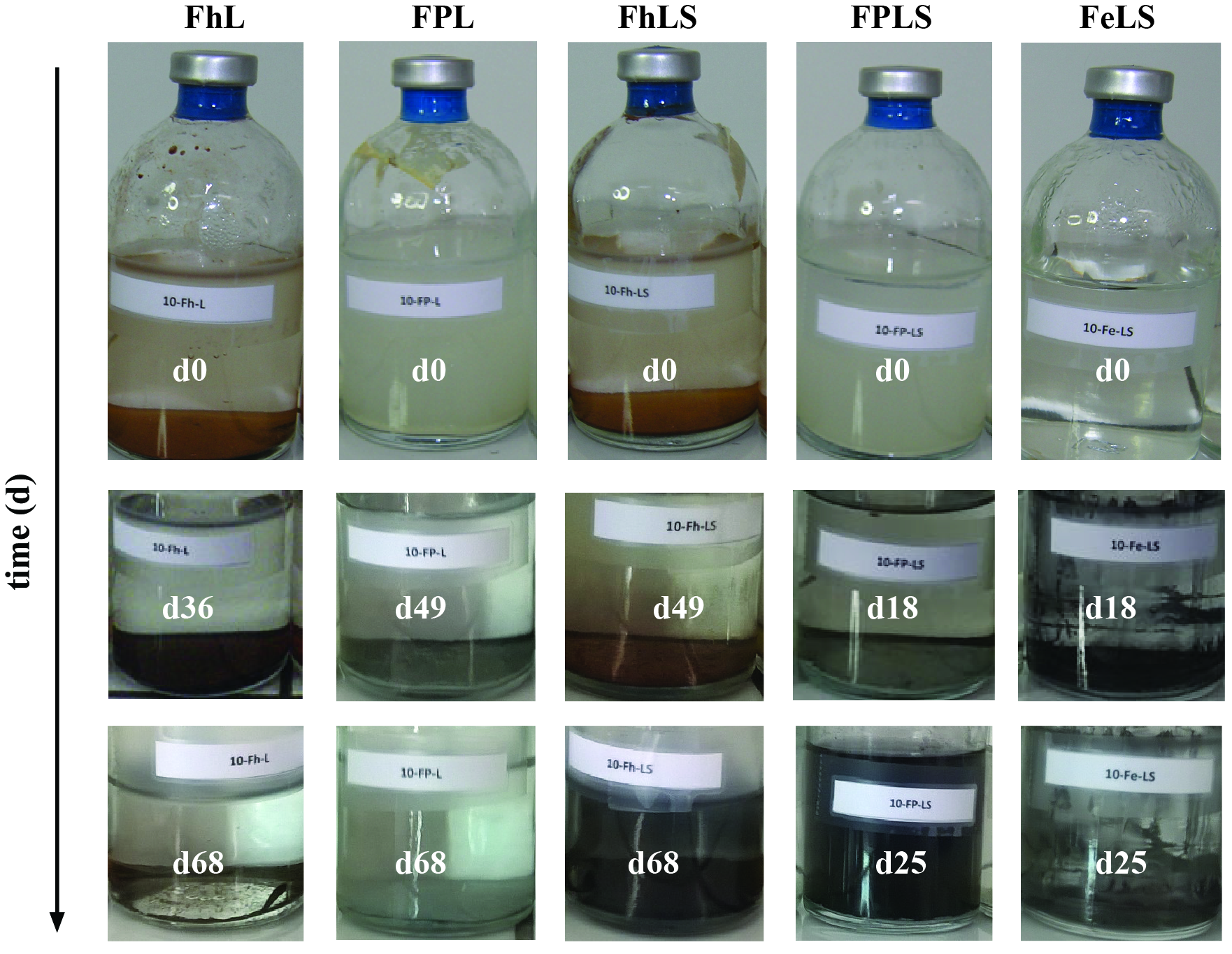

Supplement: S1 Fig — Enrichments were prepared from Lake Pavin water collected in Sept 2016 from 10 m below the oxycline with different iron phases (ferrihydrite = Fh, Fe(III)-phosphate = FP, or Fe2⁺ = Fe), without or with 10 mM added sulfate (S), and with 20 mM lactate (L) as an electron donor. (TIF) [file pone.0212787.s001.tif]
