# Supplementary material for: Microbial diversity involved in iron and cryptic sulfur cycling in the ferruginous, low-sulfate waters of Lake Pavin
Source: PLoS One. 2019 Feb 22;14(2):e0212787. doi: 10.1371/journal.pone.0212787 (PMC6386445; doi:10.1371/journal.pone.0212787)
Supplement: S1 File — (PDF) [file pone.0212787.s002.pdf]

S1 File| Fe K-edge EXAFS analysis of enrichment culture with added lactate, dissolved Fe(II) and sulfate (FeLS). left: k^3⍰(k) and right: Fourier Transform of the EXAFS signal. Black: data; red: fit. LCF analysis led to the identification of 3 components: mackinawite (88%), greigite (9%) and lepidocrocite (3%).

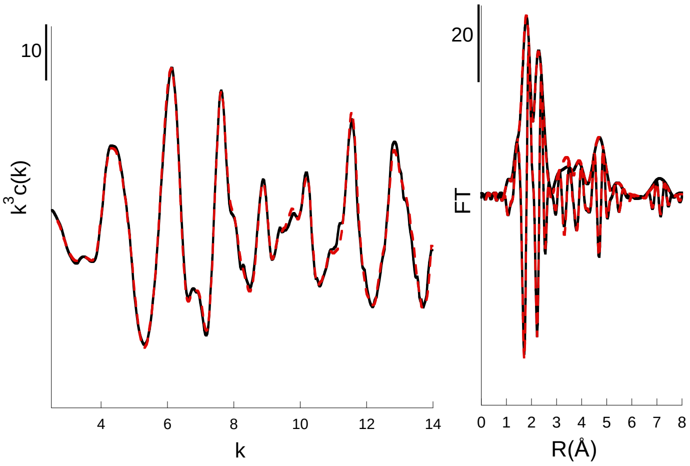

X-Ray absorption spectroscopy

Sample FeLS was analyzed by X-ray absorption spectroscopy at the Fe K-edge. Vacuum dried sample was gently grinded in an agate mortar in the glovebox, mixed with an appropriate amount of cellulose to achieve an absorption edge height (Δμx) as close as possible to 1, and compressed into pellet sealed with Kapton tape.

Fe K-edge XAS spectra were collected at 77 K (liquid N_2-cryostat) in transmission mode at the XAFS beamline (ELETTRA, Italy) using a Si(111) double-crystal monochromator. The energy was calibrated by setting the first inflection point of an Fe-foil K-edge recorded in double-transmission setup to 7112 eV. Extended X-ray Absorption Fine Structure (EXAFS) data were extracted using the program XAFS [1]. k^3-weighted EXAFS spectra (2.5 – 14 $Å^{-1}$ range) were analyzed using a Linear Combination Fit (LCF) [2,3] using the spectra from synthesized reference compounds (mackinawite, cf [4] ; greigite cf [5]; lepidocrocite cf [6]).

References

1. Winterer M. XAFS - a data analysis program for material science. Journal of Physics (Paris). 1997: 243–244.

2. Adra A, Morin G, Ona-Nguema G, Menguy N, Maillot F, Casiot C, et al. Arsenic Scavenging by Aluminum-Substituted Ferrihydrites in a Circumneutral pH River Impacted by Acid Mine Drainage. Environmental Science & Technology. 2013;47: 12784–12792. doi:10.1021/es4020234

3. Miot J, Lu S, Morin G, Adra A, Benzerara K, Küsel K. Iron mineralogy across the oxycline of a lignite mine lake. Chemical Geology. 2016;434: 28–42. doi:10.1016/j.chemgeo.2016.04.013

4. Donald R, Southam G. Low temperature anaerobic bacterial diagenesis of ferrous monosulfide to pyrite. Geochimica et Cosmochimica Acta. 1999;63: 2019–2023. doi:10.1016/S0016-7037(99)00140-4

5. Chang Y-S, Savitha S, Sadhasivam S, Hsu C-K, Lin F-H. Fabrication, characterization, and application of greigite nanoparticles for cancer hyperthermia. Journal of Colloid and Interface Science. 2011;363: 314–319. doi:10.1016/j.jcis.2010.06.069

6. Miot J, Li J, Benzerara K, Sougrati MT, Ona-Nguema G, Bernard S, et al. Formation of single domain magnetite by green rust oxidation promoted by microbial anaerobic nitrate-dependent iron oxidation. Geochimica et Cosmochimica Acta. 2014;139: 327–343. doi:10.1016/j.gca.2014.04.047
